# Bloodstream Infections Due to Wild-Type *Pseudomonas aeruginosa*: Carbapenems and Ceftazidime/Avibactam Prescription Rate and Impact on Outcomes

**DOI:** 10.3390/idr16050064

**Published:** 2024-08-27

**Authors:** Carlo Pallotto, Andrea Tommasi, Elisabetta Svizzeretto, Giovanni Genga, Giulia Gamboni, Anna Gidari, Daniela Francisci

**Affiliations:** 1 Infectious Diseases Clinic, Department of Medicine, Santa Maria della Misericordia Hospital, University of Perugia, 06132 Perugia, Italy; andrea.tommasi@ospedale.perugia.it (A.T.); elisa.svizzeretto@ospedale.perugia.it (E.S.); giovanni.genga@ospedale.perugia.it (G.G.); giualia.gamboni@ospedale.perugia.it (G.G.); daniela.francisci@unipg.it (D.F.); 2 Infectious Diseases Clinic, Santa Maria Hospital, Department of Medicine, University of Perugia, 05100 Terni, Italy; annagidari91@gmail.com

**Keywords:** *Pseudomonas aeruginosa*, bloodstream infections, BSI, carbapenem sparing, antimicrobial stewardship

## Abstract

Background. *Pseudomonas aeruginosa* is one of the major concerns among bacterial diseases even when it shows a wild-type susceptibility pattern. In 2020, EUCAST reconsidered antibiogram interpretation shifting “I” from “intermediate” to “sensible, increased exposure” with possible significant impact on antibiotic prescription. The aim of this study was to evaluate mortality in patients with *P. aeruginosa* bloodstream infections treated with antipseudomonal penicillins or cephalosporins vs. carbapenems and ceftazidime/avibactam. Methods. This is a retrospective observational study. All the patients with a bloodstream infection due to *P. aeruginosa* admitted to our hospital were enrolled. Exclusion criteria were as follows: extremely critical conditions, age <18 years, pregnancy, isolation of a strain non-susceptible to piperacillin/tazobactam and antipseudomonal cephalosporins. Patients were divided into group A (treatment with carbapenems or ceftazidime/tazobactam) and group B (treatment with antipseudomonal penicillin or cephalosporins). Results. We enrolled 77 patients, 56 and 21 in groups A and B, respectively. The two groups were homogeneous for age, sex, and biochemical and clinical characteristics at admission. All-cause in-hospital mortality was 17/56 (30.4%) and 3/21 (14.3%) in groups A and B, respectively (*p* > 0.1). In group A, in-hospital BSI-related mortality was 23.2% (13/56), while it was 14.3% (3/21) in group B (*p* > 0.1). After multivariate analysis, only the PITT score represented a risk factor for BSI-related mortality (OR 2.917, 95% CI 1.381–6.163). Conclusions. Both all-cause and BSI-related mortality were comparable between the two groups. Treatment with carbapenem or ceftazidime/avibactam did not represent a protective factor for mortality in wild-type *P. aeruginosa* BSI.

## 1. Introduction

*Pseudomonas aeruginosa* is a facultative aerobe that prefers to use oxygen as the final electron acceptor during aerobic respiration. It shows a wide—both intrinsic and acquired—resistance pattern so that it represents one of the major clinical and ecological concerns among multidrug-resistant microorganisms (MDRO) with limited treatment options; it was included in the 2024 World Health Organization (WHO) priority list [1,2]. Emergence of resistance can occur during therapy, with increased mortality [3]. Resistance can also be acquired by several mechanisms, like reduced membrane permeability, active efflux pump, target modification, and less frequently, production of degrading enzymes [4]. Prior use of carbapenem has been identified as a risk factor for carbapenem resistance, as well as male gender, intensive care unit (ICU) admission, and urinary catheterization for more than seven days [5,6]. *P. aeruginosa* is one of the most common etiologic agents in bacterial infections and, most of all, an important cause of death with a mortality rate up to 40% [7,8]. Furthermore, our country was characterised by an elevated prevalence of non-susceptible strains, as reported by the European Centre for Disease Prevention and Control (ECDC) [9] and also confirmed in real-life studies [10].

First-line treatment options include antipseudomonal penicillins and cephalosporins as well as carbapenem. When a difficult-to-treat (DTR) strain is detected, novel beta-lactam plus beta-lactamase inhibitor combinations (BLIC) and cefiderocol can be administered [11]. According to the recent ESCMID guidelines for the treatment of infections caused by multidrug-resistant Gram-negative bacilli in patients with non-severe infections or low risk of carbapenem-resistant *P. aeruginosa* isolation, considering antibiotic stewardship’s principles, antipseudomonal penicillins and cephalosporins maybe be used as monotherapy [12].

In January 2020, EUCAST, the European Committee on Antimicrobial Susceptibility Testing, released the tenth version of breakpoints table for *P. aeruginosa* [13]. In particular, EUCAST changed the meaning of “S” from “susceptible” to “susceptible, standard dose”, “I” from “intermediate” to “susceptible, increased exposure”; “wild-type” *P. aeruginosa* isolates susceptible to ceftazidime, cefepime, and piperacillin/tazobactam are now indicated as “I”. This modification represents an important paradigm shift in the interpretation of the antibiogram and in clinical practice: antibiotics indicated as “I” can be still of choice but only with an optimal pharmacokinetic/pharmacodynamic target. These targets can be achieved by (i) increasing the standard dosage (i.e., piperacillin/tazobactam 4.5 g every 6 h or cefepime 2 g every 8 h) and (ii) using at least an extended infusion (i.e., 3 h for piperacillin/tazobactam) after an initial loading dose. These changes did not affect the antibiogram of meropenem [14].

In the present observational retrospective study, we aimed to evaluate mortality in patients with *P. aeruginosa* bloodstream infections treated with antipseudomonal penicillins or cephalosporins vs. carbapenems and ceftazidime/avibactam.

## 2. Materials and Methods

Study design and participants: We consecutively enrolled all the patients admitted to our hospital from January 2020 to June 2022 with the diagnosis of BSI due to wild-type *P. aeruginosa*. 

Exclusion criteria for patients were as follows: (i) admission in extremely serious clinical condition; (ii) admission directly to the intensive care unit; (iii) death within 24 h of admission; (iv) age <18 years; (v) pregnancy; (vi) isolation of “non-wild-type” *P. aeruginosa*; (vii) treatment duration <48 h; (viii) allergy to penicillins and/or cephalosporins. We detected and evaluated 109 patients. Twelve patients were excluded because of their direct admission to the ICU in critical condition; we also excluded 3 patients who died within 24 h and 17 patients because of the isolation of antipseudomonal penicillins and cephalosporins resistant strains. Finally, 77 patients were analysed in the study.

We defined as “wild type” the strains of *P*. *aeruginosa* susceptible “standard dose” or susceptible “increased exposure” to antipseudomonal penicillins and cephalosporins, fluoroquinolons, carbapenems and beta-lactam/betalactamase inhibitor combinations. Antibiotic treatment was considered appropriate when administered intravenously at the right dosage (i.e., cefepime 2 g every 8 h, piperacillin/tazobactam 4.5 g every 6 h) and infusion time (i.e., piperacillin/tazobactam extended infusion in 3 or 4 h or continuous infusion after a loading dose).

The patients were divided into two groups based on treatment with carbapenems or ceftazidime/avibactam (group A) or with antipseudomonal penicillins or cephalosporins including ceftolozane/tazobactam (group B). We decided to include patients treated with ceftalozane/tazobactam in the last group, as it was specifically designated for *P. aeruginosa* infections, and it is therefore of primary use in this type of infection; moreover, it represents a possible treatment of choice in a carbapenem-sparing strategy with a relatively narrow spectrum when compared with beta-lactam/beta-lactamase inhibitors (BLIC). On the other hand, we included patients who underwent ceftazidime/avibactam administration in group A for its wider spectrum and under an antimicrobial stewardship point of view.

We considered only targeted treatment administered for at least 48 h for analysis. Patients were assigned to group A or group B independent of empirical therapy.

We collected demographic, epidemiological, laboratory, and clinical data in an ad hoc electronic case report form. In particular, we analysed data about gender, age, Charlson Comorbidity Index (CCI), C-reactive protein (CPR), and procalcitonin (PCT) at the time of diagnosis; to estimate clinical severity at onset, we used SOFA (Sequential Organ Failure Assessment) and PITT score. 

Outcomes: The primary outcome was all-cause and BSI-related mortality. We also evaluated risk factors for BSI-related mortality.

Statistics: Statistical analysis was performed with IBM SPSS Statistics version 23. To verify that our samples followed a normal distribution, we used Kolmogorov and Smirnov tests. To verify that the two groups were homogeneous initially, we calculated the following: median and interquartile range (IQR) for age, CCI, CPR, PCT, SOFA score, and PITT score, as these parameters constituted continuous non-Gaussian variables. Risk factors for BSI-related mortality were analysed with logistic regression and presented as odds ratio (OR) and 95% confidential interval (95% CI). *p*-values < 0.05 were considered as statistically significant. In multivariate analysis, all the variables with a *p* < 0.1 in the univariate analysis were included.

Ethics: All the patients provided a signed consent form for retrospective studies according to the local Ethics Committee recommendations. This study was conducted in line with the principles of good clinical practice and the Declaration of Helsinki. Due to the retrospective observational design of the study, formal approval was waived according to the Local Ethics Committees.

## 3. Results

The 77 patients were divided according to the administered treatment in groups A (56 patients) and B (21 patients). Table 1 summarises the characteristics of the patients at BSI onset, which were homogeneous in the two groups. In particular, no differences were detected for comorbidities at admission—especially immunocompromising conditions, SOFA score, and PITT scores at BSI onset. Primary BSIs were 18/56 (32.1%) and 9/21 (42.9%) in groups A and B, respectively (*p* > 0.1). The source of secondary BSIs were endovascular catheter-related infections 8/56 (14.3%) and 1 (4.8%), pneumonia 14 (25%) and 4 (19%), skin and skin structures infections 2 (3.6%) and 0, intra-abdominal infections 3 (5.4%) and 3 (14.3%) and urinary tract infections 11 (19.6%) and 4 (19%) in groups A and B, respectively (*p* > 0.1 for each source of infection). We detected 13/56 (23.2%) septic shock cases in group A and 4/21 (19%) in group B (*p* > 0.1). Antibiotic treatments were appropriate in 50/56 (89.3%) cases in group A and 17/21 (81%) in group B (*p* > 0.1); a monotherapy was administered in 40/56 (71.4%) and 15/21 (71.4%) cases in groups A and B, respectively (*p* > 0.1). Source control was performed in 4/35 (11.4%) cases in group A and 3/12 (25%) cases in group B (*p* > 0.1).

Table 2 describes the outcomes of the study. Mortality was assessed either as all-cause (group A: 17 [30.4%]; group B: 3 [14.3%]; *p* > 0.1) and as BSI-related (group A: 13 [23.2%]; group B: 3 [14.3%]; *p* > 0.1) Blood cultures were still positive after three days of treatment in 4/38 (10.5%) cases in group A and 4/12 (33.3%) in group B without reaching the statistical significance threshold. Notably, in 18/56 (32.1%) and 9/21 (42.9%) cases in groups A and B, respectively, control blood cultures were not performed. PCT reduction >80% within seven days was significantly more frequent in group B (9/13, 69.2%) than in group A (11/35, 31.4%) (*p* = 0.042).

To better clarify the risk factors for BSI-related mortality, we performed a univariate and multivariate analysis as shown in Table 3. In the univariate analysis, SOFA score, PITT score, and septic shock reached the threshold of statistical significance as follows: (odds ratio [OR] 1.574 [95% confidence interval (95% CI) 1.227–2.019], OR 2.015 [95% CI 1.415–2.869], and OR 8.518 [95% CI 2.474–29.324], respectively). More than an 80% reduction of PCT within seven days had OR 0.132 (95% CI 0.015–1.154) with *p* = 0.067; consequently, this parameter was included in the multivariate analysis. Notably, the following, belonging to group B, (treatment with antipseudomonal penicillins or cephalosporins or ceftolozane/tazobactam; OR 0.551 [95% CI 0.14–2.171]), did not reach the threshold of significance in the univariate analysis. In the multivariate analysis, only the PITT score was confirmed to be a risk factor for BSI-related mortality (OR 2.917 [95% CI 1.381–6.163], *p* = 0.005).

## 4. Discussion

The update of the guidelines introduced by EUCAST in 2020 has mainly changed category “I” from “intermediate” to “susceptible, increased exposure”. This new definition was linked to the PK/PD concept, and it implies that antibiotics should be administered at elevated dosages and with extended or continuous infusion to reach PK/PD targets [14]. Some studies have shown how this update could modify clinicians’ behaviour about prescriptions. In particular, a recent study analysed the impact that the EUCAST guidelines update has had on meropenem prescription, showing an increase in meropenem use after the new EUCAST breakpoint (15.2% vs. 30.2%, before and after the 2020 update, respectively). The authors also suggested that antimicrobial stewardship interventions and modifications of the reporting of antibiotic susceptibility testing could be considered to address this issue [15]. Munting and colleagues proposed masking meropenem in antibiotic susceptibility testing reports for wild-type *P. aeruginosa* isolates. This intervention was associated with a reduction in meropenem prescription (3.4% before the EUCAST update vs. 25.3% after the EUCAST update without selective reporting vs. 8.4% after the EUCAST update with selective reporting) [16].

In Italy in 2021, according to the National Institute of Health (Istituto Superiore di Sanità, ISS) report [17], the highest percentage of antibiotic resistance in *P. aeruginosa* strains was observed for piperacillin/tazobactam (23.4%), followed by ceftazidime (19.1%), fluoroquinolones (ciprofloxacin, levofloxacin, 18.6%), carbapenem (imipenem, meropenem, 16.4%), and aminoglycosides (gentamicin, amikacin, 5.7%); the indiscriminate use of antibiotics designed for MDRO may induce additional resistance; a limitation of the use of such molecules to the cases where they are strictly necessary could be important.

In our study, we compared two groups of patients with wild-type *P. aeruginosa* BSI: 56 in group A (treated with carbapenems or ceftazidime/avibactam) and 21 in group B (treated with antipseudomonal penicillins or cephalosporins including ceftalozane/tazobactam). The two groups were homogeneous both for demographic and clinical and therapeutical variables. In particular, we evaluated different types of BSI (primary vs. secondary BSI and their source of infection) and a number of patients with septic shock; no differences were detected for all these items. Moreover, the two groups were also comparable for appropriateness of antibiotic treatment and prevalence of monotherapies. 

As previously underlined, no statistically significant differences in either all-cause or BSI-related mortality were detected. These data were consistent with the literature that reported all-cause mortality of 30.6% in a prospective study [18] and a 30-day mortality of 39% in a retrospective analysis of 136 patients with *P. aeruginosa* BSI [19]. We reported a slightly lower mortality, especially in group B, probably as a consequence of the applied exclusion criteria (critically ill patients, patients directly admitted to the ICU). Our findings could be important from an antimicrobial stewardship point of view, especially highlighting that carbapenem administration in wild-type *P. aeruginosa* BSI was not associated with an improved outcome. On the other hand, mortality rate in our study remained high, especially considering that patients admitted to the ICU were excluded from the analysis according to the exclusion criteria. The elevated CCI at admission and frequency of immunocompromising conditions—about one-third of the population—might contribute to justify this finding.

We also evaluated secondary outcomes. Procalcitonin >80% reduction within seven days was significantly more frequent in group B (69.2% vs. 31.4% in group A, *p* = 0.042), while both microbiological cure (defined as blood culture negativity within three days of treatment) and hospital length of stay were comparable between the two groups.

Furthermore, risk factors for BSI-related mortality were also evaluated with logistic regression. In the univariate analysis, SOFA score, PITT score, and septic shock were statistically significant. We performed a multivariate analysis including procalcitonin >80% reduction within seven days (*p* = 0.067 in the univariate analysis). Even if only the PITT score was confirmed as a risk factor after multivariate analysis, septic shock reached a *p*-value of 0.0504, which is extremely close to the threshold of statistical significance. It could be probable that this value was a consequence of the limited sample size. Notably, belonging to group B did not represent a risk factor for mortality.

Analysing all these findings—both from our study and the literature [15]—could lead to the hypothesis that the new EUCAST guidelines could contribute to increased carbapenem and ceftazidime/avibactam prescription. On the contrary, these antibiotics should be reserved for cases with microbiological evidence of resistance to antipseudomonal cephalosporins/penicillins and ceftalozane/tazobactam. This should be desirable in order to (i) preserve carbapenem and ceftazidime/avibactam use for infections due to non-wild-type *P. aeruginosa* strains, and (ii) reduce the risk of resistance development.

This study has some limitations, in particular, its retrospective design and the small sample size. Moreover, the number of patients belonging to group A is consistently higher than group B. This is a consequence of the elevated prescribing rate of carbapenem and ceftazidime/avibactam in this setting. On the other hand, this study may help to highlight the high prescription rate of carbapenems and ceftazidime/avibactam in wild-type *P. aeruginosa* strains without a positive effect on mortality, even in patients with BSI.

In conclusion, mortality was not statistically different in the two groups despite carbapenems or ceftazidime/avibactam administration. In the antipseudomonal penicillins/cephalosporins group, PCT >80% reduction within seven days was reached significantly more frequently. Treatment with antipseudomonal penicillins or cephalosporins did not represent a risk factor for mortality. Larger studies are needed to clarify the impact of the new EUCAST definition of antibiotic prescriptions and the strategies to avoid carbapenems’ overprescription.

## Figures and Tables

**Table 1:** Characteristics of the study population.

	Study Population N = 77	Group A N = 56 (72.7%)	Group B N = 21 (27.3%)	*p*
Male gender *n* (%)	56 (72.7)	41 (73.2)	15 (71.4)	>0.1
Age *median* (*IQR*)	75 (62–82)	74.5 (61–82.25)	75 (66–80)	>0.1
CCI *median* (*IQR*)	8 (4–10)	7 (4–9)	8 (4–11)	>0.1
Immunocompromised patients, *n* (%)	25 (32.5)	18 (32.1)	7 (33.3)	>0.1
Chronic renal failure, *n* (%)	17 (22.1)	14 (25)	3 (14.3)	>0.1
CRP mg/dL *median* (*IQR*)	11.9 (7.85–19.28)	11.6 (7.98–18.03)	15.6 (4.9–20.4)	>0.1
CRP mg/dL *mean* (*SD*)	14.6 (10.1)	14.8 (10.4)	13.9 (9.5)	>0.1
PCT ng/mL *median* (*IQR*)	3.88 (1.08–22.38)	3.37 (1.26–22.78)	6.21 (0.87–21.8)	>0.1
PCT ng/mL *mean* (*DS*)	22.5 (34.4)	22.1 (33.7)	23.8 (37.5)	>0.1
SOFA score *median* (*IQR*)	4 (2–5)	4 (2–5)	3 (2–5.5)	>0.1
PITT score *median* (*IQR*)	1 (0–2)	1 (0–2.5)	1 (0–2)	>0.1
Primary BSI, *n* (%)	27 (35.1)	18 (32.1)	9 (42.9)	>0.1
Secondary BSI, *n* (%)				
- endovascular catheter-related infections	9 (11.7)	8 (14.3)	1 (4.8)	>0.1
- pneumonia	18 (23.4)	14 (25)	4 (19)	>0.1
- skin and skin structure infection	2 (2.6)	2 (3.6)	0 (-)	>0.1
- intra-abdominal infection	6 (7.8)	3 (5.4)	3 (14.3)	>0.1
- urinary tract infection	15 (19.5)	11 (19.6)	4 (19)	>0.1
Septic shock, *n* (%)	17 (22.1)	13 (23.2)	4 (19)	>0.1
Appropriate antibiotic treatment, *n* (%)	67 (87.1)	50 (89.3)	17 (81)	>0.1
Monotherapy, *n* (%)	55 (71.4)	40 (71.4)	15 (71.4)	>0.1
Source control <24 h, *n* (%)	7/47 (14.9)	4/35 (11.4)	3/12 (25)	>0.1

Abbreviations: IQR, interquartile range; SD, standard deviation; CCI, Charlson comorbidity index; CRP, C-reactive protein; PCT, procalcitonin; BSI, bloodstream infection.

**Table 2:** Outcomes.

	Study Population N = 77	Group A N = 56 (72.7%)	Group B N = 21 (27.3%)	*p*
Positive blood culture >3 days, *n* (%)	8/50 (16)	4/38 (10.5)	4/12 (33.3)	>0.1
PCT reduction >80% <7 days, *n* (%)	20/48 (41.7)	11/35 (31.4)	9/13 (69.2)	**0.042**
Hospital length of stay, days *median* (*IQR*)	25 (15–42)	27.5 (15.75–46.25)	24 (15–35)	>0.1
In-hospital mortality “all-cause” *n* (%)	20 (26)	17 (30.4)	3 (14.3)	>0.1
In-hospital mortality “BSI-related”, *n* (%)	16 (20.8%)	13 (23.2)	3 (14.3)	>0.1

Abbreviations: PCT, procalcitonin; BSI, bloodstream infection. Bold highlights the significant values.

**Table 3:** Risk factors for BSI-related mortality. Univariate and multivariate analysis.

	Univariate *Odds Ratio* (*95% CI*)	*p*	Multivariate *Odds Ratio* (*95% CI*)	*p*
Age	1.02 (0.982–1.059)	>0.1		
Male gender	0.383 (0.121–1.214)	>0.1		
CCI	1.096 (0.938–1.281)	>0.1		
SOFA score	**1.574 (1.227–2.019)**	**<0.001**	0.786 (0.414–1.489)	0.459
PITT score	**2.015 (1.415–2.869)**	**<0.001**	**2.917 (1.381–6.163)**	**0.005**
CRP at admission	1.036 (0.98–1.095)	>0.1		
PCT at admission	0.996 (0.975–1.017)	>0.1		
Immunocompromised patients	1.858 (0.6–5.756)	>0.1		
Chronic renal failure	0.438 (0.089–2.153)	>0.1		
Primary BSI	1.594 (0.519–4.902)	>0.1		
Septic shock	**8.518 (2.474–29.324)**	**0.0007**	24.903 (0.995–622.995)	0.0504
Source control <24h	0.255 (0.013–4.922)	>0.1		
Positive blood culture >3 days	2.4 (0.489–11.773)	>0.1		
PCT reduction >80% <7 days	0.132 (0.015–1.154)	0.067	1.156 (0.044–30.485)	0.931
Appropriate antibiotic treatment	1.057 (0.201–5.544)	>0.1		
Monotherapy	1.177 (0.356–3.891)	>0.1		
Group B patients	0.551 (0.14–2.171)	>0.1		

Abbreviations: 95% CI, 95% confidence interval; CCI, Charlson comorbidity index; CRP, C-reactive protein; PCT, procalcitonin; BSI, bloodstream infection. Bold highlights the significant values.

## Data Availability

Data will be available after an appropriate request to the corresponding author.
